# Extraction and Characterization of Microplastics from Portuguese Industrial Effluents

**DOI:** 10.3390/polym14142902

**Published:** 2022-07-17

**Authors:** Solange Magalhães, Luís Alves, Anabela Romano, Bruno Medronho, Maria da Graça Rasteiro

**Affiliations:** 1 CIEPQPF, Department of Chemical Engineering Pólo II–R. Silvio Lima, University of Coimbra, 3030-790 Coimbra, Portugal; solangemagalhaes@eq.uc.pt; 2 MED–Mediterranean Institute for Agriculture, Environment and Development, Faculdade de Ciências e Tecnologia, Campus de Gambelas, Universidade do Algarve, Ed. 8, 8005-139 Faro, Portugal; aromano@ualg.pt; 3 FSCN, Surface and Colloid Engineering, Mid Sweden University, SE-851 70 Sundsvall, Sweden

**Keywords:** microplastics, Portugal, resin, pharmaceutical, PVC, paint, wastewater treatment plant

## Abstract

Microplastics (MPs) are contaminants present in the environment. The current study evaluates the contribution of different well-established industrial sectors in Portugal regarding their release of MPs and potential contamination of the aquifers. For each type of industry, samples were collected from wastewater treatment plants (WWTP), and different parameters were evaluated, such as the potential contamination sources, the concentration, and the composition of the MPs, in both the incoming and outcoming effluents. The procedures to extract and identify MPs in the streams entering or leaving the WWTPs were optimized. All industrial effluents analysed were found to contribute to the increase of MPs in the environment. However, the paint and pharmaceutical activities were the ones showing higher impact. Contrary to many reports, the textile industry contribution to aquifers contamination was not found to be particularly relevant. Its main impact is suggested to come from the numerous washing cycles that textiles suffer during their lifetime, which is expected to strongly contribute to a continuous release of MPs. The predominant chemical composition of the isolated MPs was found to be polyethylene terephthalate (PET). In 2020, the global need for PET was 27 million tons and by 2030, global PET demand is expected to be 42 million tons. Awareness campaigns are recommended to mitigate MPs release to the environment and its potential negative impact on ecosystems and biodiversity.

## 1. Introduction

Microplastics (MPs) have been gaining increasing awareness after several reports regarding “garbage patches” in the world’s oceans [1]. Similarly to climate changes and persistent organic pollutants, plastic residues are also perfect examples of the human capacity to significantly affect ecosystems and biodiversity on a global scale. MPs have been detected and identified in many different environments, such as aquatic media, ground waters, landfill leachate, wastewater, and sewage sludge [2,3,4]. MPs have also been detected in remote areas, including polar regions, such as Antarctica of the southern ocean and on the deepest parts of the ocean at the Mariana Trench, North Pacific gyre, and south pacific islands, with particularly high concentrations [5,6,7]. Even in the world´s highest hills, the Tibetan plateau, MPs have been detected within its rivers and lakes [8]. Wastewater treatment plants (WWTPs) can mitigate the spreading of MPs by retaining part of them (larger dimensions) with screens and filters [9,10,11]. In WWTPs, ca. 78–98% of MPs can be removed after primary treatment, while the secondary treatment is responsible for a smaller decrease, ca. 7–20% [12,13]. However, it has been shown that MPs can pass through the WWTP, flowing into the aquatic media and accumulating in the environment [14,15]. Furthermore, the aggregation of MPs with other suspended solids in wastewaters induces their accumulation in sewage sludge [16,17]. The abundance of MPs in wastewaters and sewage sludge varies depending on the time of the day, season, and type of wastewaters (i.e., domestic or industrial). Urbanization and industrialization are among the main reasons for the observed increasing contamination of MPs. In this respect, daily industrial discharges of different manufacturing industries, such as paint, pharmaceutical, textile, resins, cosmetics, etc., can have a significant impact on the total amount of MPs released [18,19]. MPs can then easily enter the food chain. Their chemical composition is complex and includes different polymeric substances and additives (e.g., flame-retardants, dyes, plasticizers, and UV-inhibitors) that may impart toxicity to living beings, or act as adsorbent agents for other harmful organic pollutants [1,19]. 

It is generally recognized that the most important contamination sources of MPs arise from the textile and paint/coating industries [1,20,21,22]. Textiles are flexible materials that mainly consist of natural and/or synthetic fibres. Today´s technology allows the manufacturing of several types of fabrics, which can be made of pure fibres (e.g., viscose, polyester, etc.) or mixtures of fibres (e.g., cotton–polyester). Mixing different types of fibres helps to enhance the physical characteristics of the final material (e.g., elasticity, strength, durability) and eventually lowers the price [22]. On the other hand, the paint/coating industries’ contamination focuses mainly on alkyd ship paint resins and poly (acrylate/styrene) from fibreglass resins. In this case, MPs are concentrated at the surface microlayer of the ship [1].

In Portugal, there are about sixty-nine thousand manufacturing companies, 17% being chemical-related industries and 5% dealing with textiles. It has been estimated that ca. 72% of the waste found in Portuguese industrial areas and estuaries are MPs [23,24,25,26]. Industrial spillages, emissions from road traffic, atmospheric deposition, wind-blown debris from littering or loss during waste disposal, and the degradation of larger plastic debris directly in the aquatic media may further contribute to MPs contamination in aquatic ecosystems [27].

The efficient separation and identification of MPs in the inflow and outflow effluents of WWTPs is very desirable but technically challenging due to the complexity of these streams [15,28]. Typically, the MPs detection involves three main steps but, so far, the methods used in each stage have not been standardized [29].

The MPs separation process is usually performed with a series of sieves or filters of different mesh/pore sizes in which the collected effluent is forced to pass [12]. Afterwards, the material on each sieve can be washed with distilled water and deposited into glass vials. However, the procedure has several problems, such as the clog of the sieves with organic load [30]. Ziajahromi et al. (2017) developed a method for MPs separation from wastewater effluents [31]. This method involves a high-volume sampling device with multiple mesh screens to collect a wide size range of MPs from wastewater effluents. This process is combined with an efficient sample processing procedure using organic matter digestion, density separation, and staining to identify and eliminate the non-plastic particles [31]. The efficiency of the technology is highly satisfactory as the capture of MPs ranged from 92% for the 25 µm mesh screen to 99% for the 500 µm mesh screen. This shows that the sampling device is suitable to capture MP with a wide range of particle sizes. However, the sieve-based separation presents some limitations, also highlighted in the National Ocean and Atmospheric Administration (NOAA) norm, which are related to the MP morphology and size. For example, microfibres, have a high aspect ratio and thus can be either retained horizontally in the sieve or pass longitudinally through the sieve holes.

Another procedure to extract MPs of different nature (i.e., PP, PVC, and PET) was described by Nuelle et al. (2014), where MPs were extracted from sediments using a two-step approach. The authors suggest that the developed method is suitable to monitor MPs in marine sediments [32]. As mentioned, the technique consists of two main steps: (i) fluidisation of sediments in a saturated NaCl solution and (ii) subsequent flotation of MPs in a high-density NaI (aqueous solution). With this two-step approach, it was possible to efficiently extract common synthetic macromolecules, including high-density polymers from contaminated sediments. Compared with other systems based on flotation in high-density salts, this method is more advantageous, due to the availability of materials used, and the simplicity of the equipments, rendering the whole process cost-effective [32].

A slightly different method was proposed by Besley et al. (2017) who used a fully saturated salt solution of NaCl combined with filtration for the extraction of MPs [33]. The extraction of the MPs is accomplished by density separation followed by drying with a saturated salt solution. The supernatant is afterwards submitted to vacuum filtration and the filter membranes containing the MP particles are examined by stereo-microscopy. This method allows for the quantification of MPs in the range of 0.3–5 mm [33].

Wei et al. (2020) used sieves to remove materials larger than 5 mm in length. Then, H_2_O_2_ is added to the filtrate at 80 °C to degrade the organic matter. After volume reduction, plastic components are separated from non-plastic materials via density separation (40% CaCl_2_ solution). Finally, the samples are centrifuged at 5000 rpm and, the supernatant particles are vacuum filtered using a glass filter [34]. The drawback of this laborious process is that MPs with a higher density can precipitate during the centrifugation process thus underestimating the global MP quantification.

In general, all the methods used to separate the MPs from the aqueous medium are based on the National Ocean and Atmospheric Administration (NOAA) norms for processing samples. Note that the standard procedure was designed for samples collected in marine environments. However, due to the complex matrix of wastewater samples and the presence of numerous particles of organic nature and other compounds of different sources, this procedure is not considered suitable for other non-marine samples. This method involves the digestion of organic matter using H_2_O_2_ in the presence of an aqueous ferrous solution (Fe(II)) as a catalyst. The digestion step is usually followed by a separation stage, which uses NaCl (aq) or ZnCl_2_ (aq) to increase the density of the liquid phase. Through this procedure, the low-density MPs tend to float, while the high-density particles settle at the bottom. Then, the liquid phase is filtered through mesh sizes varying from 0.7 to 125 μm [35].

Despite the low concentrations of MPs typically reported for treated effluents, the number of MPs released to recipient waters can still be very high over relevant temporal scales [36]. Therefore, the present work is focused on the evaluation of the release of MPs in the treated effluents of WWTPs and the main goal is to elucidate the role of different Portuguese industries as potential sources of MPs contamination to ecosystems. To do so, different effluents from the paint, resin, pharmaceutical, textile, and PVC industries were collected, and the MPs were analyzed regarding their concentration and type, after implementing suitable separation and cleaning protocols. The relationship between the different industries and MPs predominancy is discussed. Another relevant point that should be highlighted is that the procedure suitable for extracting MPs was based on a generic method, but it had to be adapted to each effluent, based on the specificities of each sample (i.e., different organic or inorganic fractions, presence of other contaminants, etc).

## 2. Materials and Methods

### 2.1. Materials

Five different Portuguese industrial effluents (i.e., paint, resin, textile, pharmaceutical, and PVC) were collected and analysed before and after the company’s in-house WWTP. Ultra-pure water (UPW) and ultra-filters (pore size < 2 mm) were used for all extraction and characterization steps. The H_2_O_2_ (30%) was purchased from Greendet, Portugal. KOH was obtained from LabKem, Spain. HCl (37%) was purchased from Honeywell, Portugal. To minimize the potential contamination by other agents, all reagents were filtered before use with 0.45 μm syringe PTFE filters. The anionic surfactant sodium alkylbenzenesulfonate (99%) was obtained from Sigma-Aldrich (St. Louis, MO, USA).

Sample filtration was performed using a vacuum filtration unit made of glass (FiltresRS par Jean-Pierre D., France). The glass fibre filters (pore size 1–2 μm) were burned at 250 °C for 30 min before use. 

### 2.2. Sample Preparation and Extraction of the Microplastics

The protocols commonly used for MPs extraction and analysis often require long digestion times and different preparation steps [37]. Our approach involved an initial alkaline treatment, 10% KOH (aq) for 12 h at 50 °C, to remove organic matter [38]. After the alkaline treatment, acid-based digestion was employed, consisting of 20% HCl (aq) solution for 15 min (adapted from Nuelle et al., 2014 [32]). This treatment digests any biological residues or inorganic materials, such as calcium carbonate. After the basic and acidic digestions, the samples were filtered with 1–2 μm glass filters. The MPs were then separated via their density differences with a concentrated NaCl (aq) solution, with a density of 1.2 g/cm^3^. Finally, after separated, the MPs were cleaned with H_2_O_2_ and ethanol, to remove the presence of any hypothetical microorganisms from their surfaces. It is important to point out that this protocol was adjusted depending on the type of effluent. That is, depending on the effluent source, the amounts of organic, inorganic compounds and/or other contaminants may vary, thus requiring special care. For instance, in the case of the pharmaceutical effluent, a 1% anionic surfactant solution was used before the alkaline digestion to enhance filtration and MPs separation.

### 2.3. Characterization and Quantification of Microplastics

The size and shape of MPs were analysed in an Olympus BH-2 KPA optical microscope (Olympus Optical Co., Ltd., Tokyo, Japan) equipped with a high-resolution CCD colour camera (ColorView III, Olympus Optical Co., Ltd., Tokyo, Japan). The size was further accessed by laser diffraction spectroscopy (LDS) in a Malvern Masterziser 2000 (Malvern Instruments, Malvern, UK). The effluent samples were loaded in the equipment dispersion unit until a fixed level of obscuration (i.e., 6–7% guarantees good signal-to-noise quality). The LDS tests were carried out by setting the pump speed to 1500 rpm, corresponding to an average shear rate of 334 s^−1^. 

The charge density of MPs was evaluated by measuring the zeta potential of the effluent samples in a Zetasizer NanoZS equipment (ZN 3500, Malvern Instruments, Malvern, UK), in the electrophoretic light scattering mode. In brief, the samples were gently transferred to a folded capillary zeta cell, the presence of air bubbles was visually checked and then the cell was left to equilibrate at 25 °C before starting the assay. Each sample was scanned 3 times, each time with 12 runs. The results were processed using the Zetasizer Nano Software 7.13 (Malvern Instruments).

The MPs quantification was performed by gravimetry, weighing the filter before starting the filtration process (after burning at 250 °C) and after filtration and being oven-dried at 105 °C for 8 h. The MPs amount was estimated by the mass difference in 100 mL of each effluent type.

### 2.4. Microplastics Identification

The identification of the polymer types present in the isolated MPs was accessed by Fourier transform infrared spectroscopy (FTIR). The obtained spectra of the MPs before and after the company’s in-house WWTP treatment were obtained and compared with an existing FTIR library. The FTIR spectra were obtained using an ATR-FTIR (Perkin–Elmer FT-IR spectrometer, Waltham, MA, USA) with a universal ATR sampling accessory, between 500 cm^−1^ and 4000 cm^−1^, resolution of 4 cm^−1^ and applying 128 scans. 

Fluorescence microscopy was also employed to characterize the isolated MPs, using pyrene as the fluorescent probe. Pyrene has demonstrated excellent sensitivity towards surface-modified carboxyl particles [39]. In brief, 200 μL of the pyrene stock solution (1 mg/mL) were diluted into 20 mL of milli-Q water and poured into the filtration funnel. To achieve a reliable stanning, the solution was allowed to be in contact with the filter disc for ca. 15 min. After this period, the excess solution was removed by vacuum filtration and the filter disc was placed in the filter holder. Samples were imaged at room temperature in a fluorescence microscope (Olympus BX51M), equipped with a 100 × objective lens, a filter set type U-MNU2 (360–370 nm excitation and 400 nm dichromatic mirror) and a UV–mercury lamp (100 W Ushio Olympus, Olympus Optical Co., Ltd., Tokyo, Japan). Images were obtained through a video camera (Olympus digital camera DP70, Olympus Optical Co., Ltd., Tokyo, Japan) and analysed with an image processor (Olympus DP Controller 2.1.1.176, Olympus DP Manager 2.1.1.158, Olympus Optical Co., Ltd., Tokyo, Japan). 

### 2.5. Statistical Analysis

Statistical analysis was performed using one-way ANOVA (α = 0.05) to evaluate significant differences between the zeta potential of the effluents studied.

## 3. Results

MPs were not readily considered as potential sources of contamination and, for a long time, most samples were analysed with little to no concern about their potential impact. However, with the awareness that MPs are virtually everywhere, contaminating different ecosystems and having potential toxic effects on animals and plants, new strategies must be developed to understand their hypothetical impact and mitigate it. This motto has been the driving force for this study. A total of five effluents from different Portuguese industries (i.e., paint, resin, pharmaceutical, PVC, and textile) were analysed to infer their contribution to the MPs release and pollution of aquifers. Effluents are strongly dependent on the source and thus differ from each other, since these are complex mixtures of several pollutants, including synthetic chemicals, hydrocarbons, acrylic polymers, inorganic compounds, and heavy metals [40]. Effluents from the resin industry are usually composed of acrylic polymers and inorganic compounds, such as calcium carbonate, plasticizers, etc., while PVC effluents are generally composed only of polymers and additives that are incorporated during the polymerization process for PVC morphology control [41]. On the other hand, effluents from the pharmaceutical industry are typically heterogeneous mixtures of polymers, surfactants, antibiotics, and organic contaminants, among others [42]. Similarly, textile-based effluents can contain numerous toxic compounds, such as nonylphenol ethoxylates, benzothiazole, dyes, etc. [43]. 

**Figure 1:**
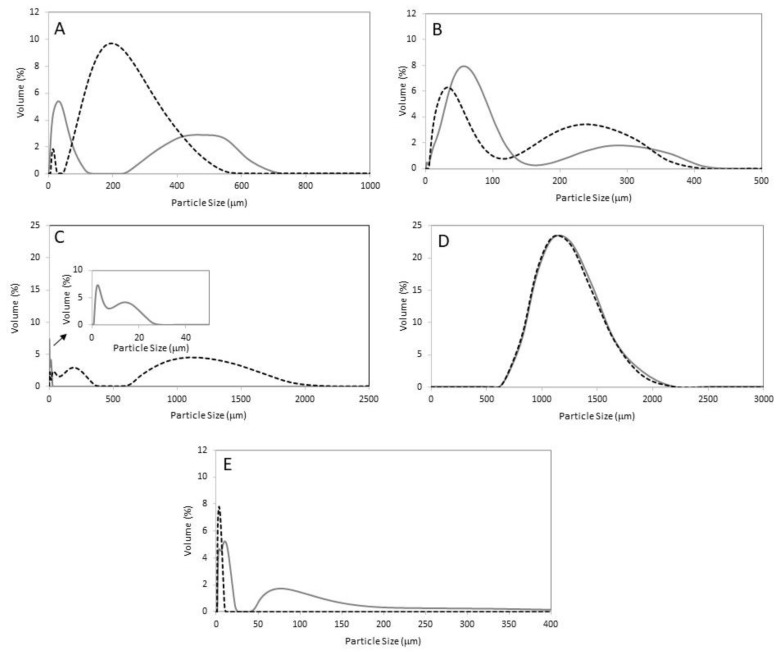
LDS of the different effluents as collected, before (solid grey lines) and after (dashed black lines) the in-house WWTP of each industry: (**A**) resins; (**B**) paint; (**C**) pharmaceutical; (**D**) PVC, and (**E**) textil. The assays were performed at 25 °C.

The particle size of the effluents was characterized in samples obtained before and after each in-house WWTP treatment (Figure 1).

As can be observed in Figure 1, all effluents have two well-defined size populations of the particles. The exception is the effluent from the PVC industry (Figure 1D), where the size distribution is the same before and after the in-house WWTP. In the effluent from the textile industry (Figure 1E), the in-house WWTP treatment seems to remove the larger particles, thus increasing the volume% of the smaller particles. The effluents with larger particles are the ones coming from the pharmaceutical (Figure 1C) and the PVC (Figure 1D) industries, while the smallest particles were detected in the effluent from the textile industry. Except for the PVC effluent, the in-house WWTPs decrease the particle size. Therefore, it is reasonable to assume that these treatments are efficiently removing the bigger particles shifting the population towards smaller sizes. 

The average charge density of the collected effluents was estimated via zeta potential, as summarized in Table 1. Again, samples were analysed before and after each in-house WWTP.

All effluents showed significant differences in zeta potential, except the effluent of resin and PVC industries, before treatment. For the effluents after treatment, all are significantly different in zeta potential. The results of the one-way ANOVA tests are summarized in Table S1. Larger differences are observed comparing the inflow and outflow effluents for the same industry.

Overall, all the effluents presented a negative charge density before and after treatment, except the pharmaceutical effluent before treatment, probably due to the presence of cationic polymers in the solution (e.g., cationic polyacrylamides). Therefore, at environmentally relevant pH and above, the MPs are generally negatively charged and consequently so is the effluent [44]. 

Following the procedure described in the experimental section, the MPs from the different effluents (before and after the in-house WWTP) were extracted, cleaned, and quantified (Table 2).

Although all in-house WWTP decreased the % of MPs released, significant amounts of MPs are still liberated and potentially contaminating different ecosystems. Among the different sectors analysed, the textile industry is surprisingly observed to contribute the least. Yet, some studies have shown that synthetic fibres are the dominant type of polyester MPs detected in aqueous media, sediments, and various organisms [43,45,46]. This can be reasoned by the massified and extensive domestic and industrial washing cycles continuously releasing MPs into wastewaters. It should be highlighted that the textile WWTP shows a lesser ability to retain MPs particularly due to their smaller size (see Figure 1). 

**Figure 2:**
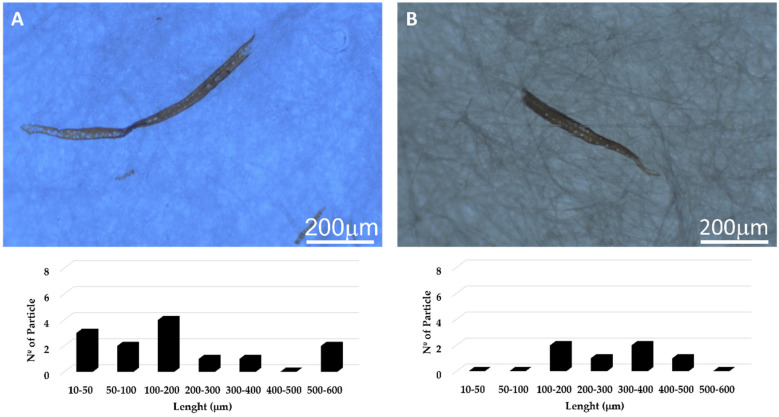
Optical micrographs and size distributions of the MPs before (**A**) and after (**B**) applying the MPs extracting procedure developed in this work to the resin effluent. Note that the data regarding the other effluents can be found in the Supplementary Information section.

The shapes of MPs recovered from the different effluent samples were mainly elongated fibres and fragments. Fibres constitute the majority as this shape allows an easier diffusion through the in-house WWTP filters. The fragments, in addition to being easily retained in the WWTP filters, can form larger aggregates with the flocculating agents used and thus become more accessible for trapping in the in-house WWTP. In the samples studied, the observed sizes range from 10 µm to 500–600 µm (Figure 2); it has been reported that 80% of the MPs fall within a size range of 125–500 µm [47]. Smaller MPs have also been detected in this work, which can be either due to the specific nature of the effluents tested and/or partial MPs degradation during the cleaning procedure. The alkaline, acidic, and oxidizing treatments used may further fractionate the MPs [48,49]. To infer the effect of our developed “cleaning” treatment on the MPs structure, the samples were analysed before and after applying it. In Figure 2, a typical example of the MPs shape and size distributions is shown for the resin effluent. Similar qualitative data was obtained for the other effluents (Figure S1). Although the trends are not fully clear, three main conclusions can be drawn: (i) the lowest size reported (i.e., 10–50 µm) tends to decrease upon “cleaning” the samples (this is particularly striking in the resins effluent); (ii) the bigger size reported (i.e., 500–600 µm) decreases after “cleaning” the effluents; (iii) the intermediate sizes (i.e., 50–500 µm) tend to increase their number density, most likely as a result of the fractionation of the bigger fibres. Therefore, this analysis shows that the cleaning procedure (applied to remove unwanted inorganic and organic compounds) is not innocuous and can, in fact, affect the MPs shape and size. Although often neglected in the literature, any reliable analysis should not rule out its contribution. 

Besides the shape and size, it is also important to study the predominant type of MPs in each effluent. Based on the composition of the plastics, they can be classified into two main categories: (i) thermosetting plastics, such as polyimides and bakelite, and (ii) thermoplastics, such as polyethylene terephthalate, polypropylene, and polyethylene [50]. Typically, thermoplastic materials have a highly cross-linked structure, consisting of a three-dimensional network of covalently bonded atoms, displaying remarkable physical properties, such as high thermal stability, high rigidity, high dimensional stability, resistance to creep or deformation under load and high electrical and thermal insulating capacity [51]. Moreover, thermoplastics, which are far more abundant, can be heated and melted and then cooled and reformed into new shapes, thereby allowing many of them to be recycled [52]. On the other hand, the majority of thermosetting plastics are permanently set and cannot be melted and reformed. This irreversible chemical change does not allow the recycling of thermosetting plastics. 

**Figure 3:**
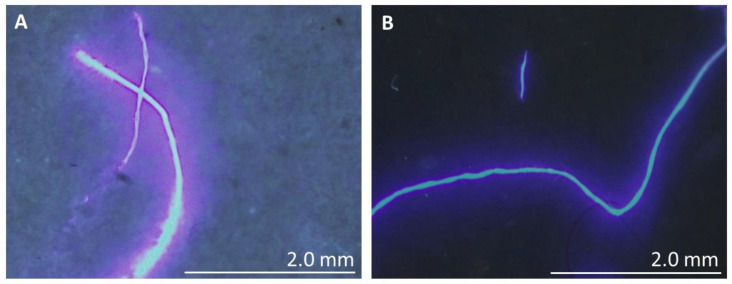
Fluorescence micrographs of the MPs before (**A**) and after (**B**) applying the cleaning procedure developed in this work to the PVC effluent. Samples were stained with pyrene (see the experimental section for details). Note that the data regarding the other effluents can be found in the Supplementary Information section.

A useful recent technique to infer the chemical composition of the MPs is fluorescence microscopy [43,53]. The fundamentals are fairly easy to understand; samples are stained with suitable dyes, such as pyrene, and, depending on the composition, they will fluoresce (decay after excitation with UV light) at different wavelengths. In practice, this means that the colour observed in the fluorescence microscope can be correlated to the particle composition. Pyrene is particularly sensitive to the different MPs polarities, thus being a suitable dye [34,38]. In Figure 3, typical fluorescent micrographs of the MPs from the PVC effluent are shown. Depending on the effluent type (and MPs composition) different emissions can be observed (Figure S2). 

As can be seen in Figure 3 and Figure S2, the steady-state fluorescence emission spectra of pyrene-stained samples mostly present MPs with green, blue, and red colours, which can be assigned to high-density polyethylene (HDPE), polyethylene (PE), and polyethylene terephthalate (PET), respectively [43]. In the effluents from the paint, resin, PVC, and textile industries (see Figure 3 and Figure S2), the presence of PET can be mainly observed. Only in the case of the effluent from the pharmaceutical industry, was the presence of HDPE, PE and PET MPs possible to observe through the same measurements (Figure S2E,F). The worldwide production of plastics, according to their polymer composition, is as follows: 36% polyethylene (PE), 21% polypropylene (PP), <10% polyethylene terephthalate (PET), <10% polyurethane (PUR), and <10% polystyrene (PS) [47]. Therefore, the largest used plastic types worldwide are in perfect agreement with most of the MPs observed in this work.

**Figure 4:**
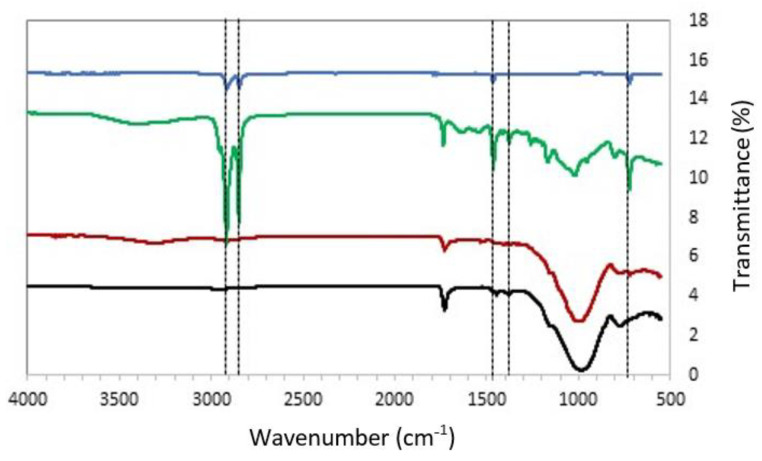
FTIR spectra of the MPs from the pharmaceutical effluent before (red curve) and after (green curve) the in-house WWTP. The FTIR spectra from the filter (black curve) and neat PE (blue curve) are also shown. The main vibrational modes are highlighted with the dashed vertical lines and their assignment are discussed in the main text. The FTIR spectra from the remaining effluents can be found in the Supplementary Information section [54,55].

ATR-FTIR was also used to complement the fluorescent data and infer the MPs composition. This technique is usually suitable only for clean isolated particles that are big enough to be placed on the ATR crystal [48]. However, under optimized conditions, it was possible to identify the composition of the most prevalent types of MPs. In Figure 4, typical FTIR spectra are shown for the MPs from pharmaceutical effluent, before and after being submitted to the in-house WWTP procedure. The spectrum of neat PE is also shown for reference. The pharmaceutical effluent shows vibrational modes that are also characteristic of model PE, such as the rocking deformation, wagging deformation, and asymmetric stretching of CH_2_ groups. It is also possible to identify vibrational bands that can suggest the presence of PP. In particular, the band at 600 cm^−1^ can be attributed to the CH wagging mode, the band at 844 cm^−1^ is assigned to C-CH stretching, at 961 cm^−1^ is assigned to the trans CH wagging, while the band at 1240–1252 cm^−1^ is assigned to CH rocking vibration and the band at 2890–2950 cm^−1^ can be attributed to the CH_2_ asymmetric stretching of PP. Similar assignments can be done for the remaining effluents (see Figure S3 for details). Another striking observation for all samples tested (see Figure S3 for the remaining effluent types) is that after each in-house WWTP treatment, the intensity of the FTIR bands decreases. Although FTIR is not a truly quantitative method, these results suggest that the in-house WWTP reduce the number of MPs in the treated effluent, which agrees with the gravimetric quantifications presented in Table 2. Overall, FTIR complements and supports the fluorescent microscopy data showing that the main compounds present in the MPs are PE and PP.

In brief, it was possible to detect MPs in the studied effluents from different industries being prevalent the MPs from the plastic types widely used, such as PE, PP, and PET. The contamination with MPs is a real problem, and the WVTP treatments used nowadays, at least in the industries analysed, are not sufficiently robust to remove/retain all MPs. Thus, it is urgent to develop methods that allow improved efficiency and higher removal yields of these contaminants from the industrial effluents. 

## 4. Conclusions

Presently, MPs are a well-distributed contaminant virtually all around the world, whose potential negative impact on different ecosystems is still hard to evaluate. It has been shown that the amount of MPs varies significantly depending on the source, but it is particularly relevant in industrial effluents. The inland Portuguese case here explored is no exception. Nevertheless, the predominant MPs type in all the industrial areas studied was PET, which agrees with the fact that it is the most common and widely used thermoplastic polymer. It is also striking, as can be gleaned from this work, that accurate MP extraction and quantitative and qualitative characterizations require special care for a reliable and free-of-objects analysis. This work also revealed that, in the Portuguese case, the textile industry is the sector that does not contaminate (regarding MPs release) the most; the larger number of MPs detected come from pharmaceutical and paint effluents. Further studies are required to understand whether these results reflect the real Portuguese scenario or sporadic technical issues in the in-house WWTP of these industries. This hypothesis should not be ruled out as occasional malfunction problems may compromise an overall appreciation. Regardless of the case, we are confident in stating that the in-house WWTP does not reveal a satisfactory efficiency regarding MPs retention. Serious future efforts should be considered to efficiently mitigate MPs release, especially when, at this stage, little is known about their real impact on the ecosystems. This work expects to bring awareness to this potential threat, as it is important that industries do not neglect their treated effluents as a significant possible MP disseminator source, despite having a WWTP in their facilities. We suggest that this study or other similar analyses should be extended to other countries to understand the trends on a global scale and the real dimensions of the MP threat.

## Figures and Tables

**Table 1:** Zeta potential of the different effluents, before and after each company in-house WWTP.

*Industries*	*Before Treatment (mV)*	*After Treatment (mV)*
*Resin*	−13.13 (±0.90) *	−4.93 (±0.24)
*Paint*	−3.94 (±0.21)	−6.20 (±0.18)
*Pharmaceutical*	8.47 (±1.07)	−9.90 (±0.70)
*PVC*	−12.30 (±0.56) *	−7.89 (±0.08)
*Textile*	−28.30 (±1.47)	−31.13 (±1.40)

* values marked with the same symbol mean no statistical difference (*p* < 0.05).

**Table 2:** Gravimetric quantification of MPs before and after each company’s in-house WWTP. An estimative of the MPs amount released per ton of effluent is also presented.

	Before in-House WWTP (g MPs/100 mL Effluent)	After in-House WWTP (g MPs/100 mL Effluent)	MPs Released after in-House WWTP (g MPs/Ton Effluent)
Resins	0.044 ± 0.020	0.004 ± 0.002	41.66
Paint	0.172 ± 0.095	0.009 ± 0.004	89.01
Pharmaceutical	0.004 ± 0.003	0.002 ± 0.002	24.69
PVC	0.029 ± 0.001	0.007 ± 0.006	70.58
Textile	0.002 ± 0.002	0.002 ± 0.002	15.65

## Data Availability

The data presented in this study are available on request from the corresponding author.

## Supplementary Materials

The following supporting information can be downloaded at: https://www.mdpi.com/article/10.3390/polym14142902/s1, Figure S1: Optical micrographs and size distributions of the MPs before (left column) and after (right column) applying the cleaning procedure developed in this work to the resin (A,B), paint (C,D), pharmaceutical (E,F), PVC (G,H) and textile (I,J) effluents. Note that below each representative optical micrograph the correspondent size distributions can be found. Figure S2: Fluorescent micrographs of the MPs before (left column) and after (right column) applying the cleaning procedure developed in this work to the PVC (A,B), resin (C,D), paint (E,F), pharmaceutical (G,H) and textile (I,J) effluents. Figure S3: FTIR spectra of the MPs from the (A) resin, (B) paint, (C) PVC and (D) textile effluents before (red curves) and after (green curves) the in-house WWTP. The FTIR spectra from the filter (black curve) is also shown. The main vibrational modes are highlighted with the dashed vertical lines and its assignment is discussed in the main text and bellow. Table S1: Statistical treatment of charge results. The symbol (-) indicates significant differences in the effluents studied, and (*) indicates no significant differences from one-way ANOVA tests (*p* ≤ 0.05).
