# Supplementary material for: Extraction and Characterization of Microplastics from Portuguese Industrial Effluents
Source: Polymers (Basel). 2022 Jul 17;14(14):2902. doi: 10.3390/polym14142902 (PMC9318256; doi:10.3390/polym14142902)
Supplement: Supplementary file 1 [file polymers-14-02902-s001.zip › polymers-1756421-supplementary_v2.pdf]

Supporting Information

Extraction and characterization of microplastics from Portuguese industrial effluents

Solange Magalhães, Luís Alves, Anabela Romano, Bruno Medronho, Maria da Graça Rasteiro

Table S1: Statistical treatment of charge results. The symbol (-) indicates significant differences in the effluents studied, and (*) indicates no significant differences from one-way ANOVA tests ($p \leq 0.05$).

Before treatment			After treatment		
resin	textile	-	resin	textile	-
resin	paint	-	resin	paint	-
resin	pharmaceutical	-	resin	pharmaceutical	-
resin	PVC	*	resin	PVC	-
textile	paint	-	textile	paint	-
textile	pharmaceutical	-	textile	pharmaceutical	-
textile	PVC	-	textile	PVC	-
Paint	pharmaceutical	-	paint	pharmaceutical	-
paint	PVC	-	paint	PVC	-
pharmaceutical	PVC	-	pharmaceutical	PVC	-

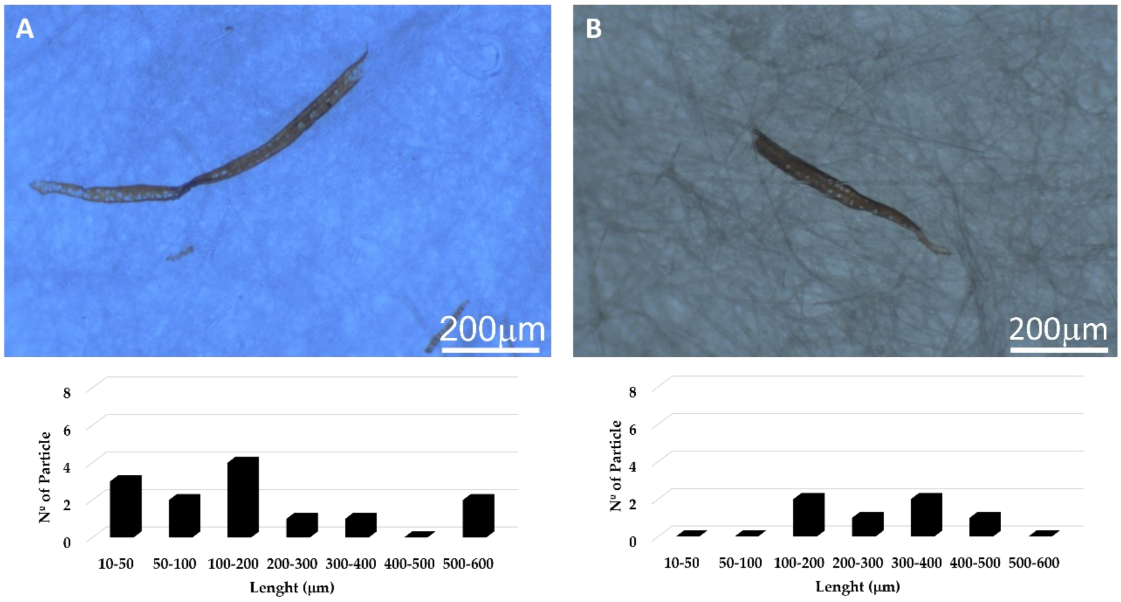

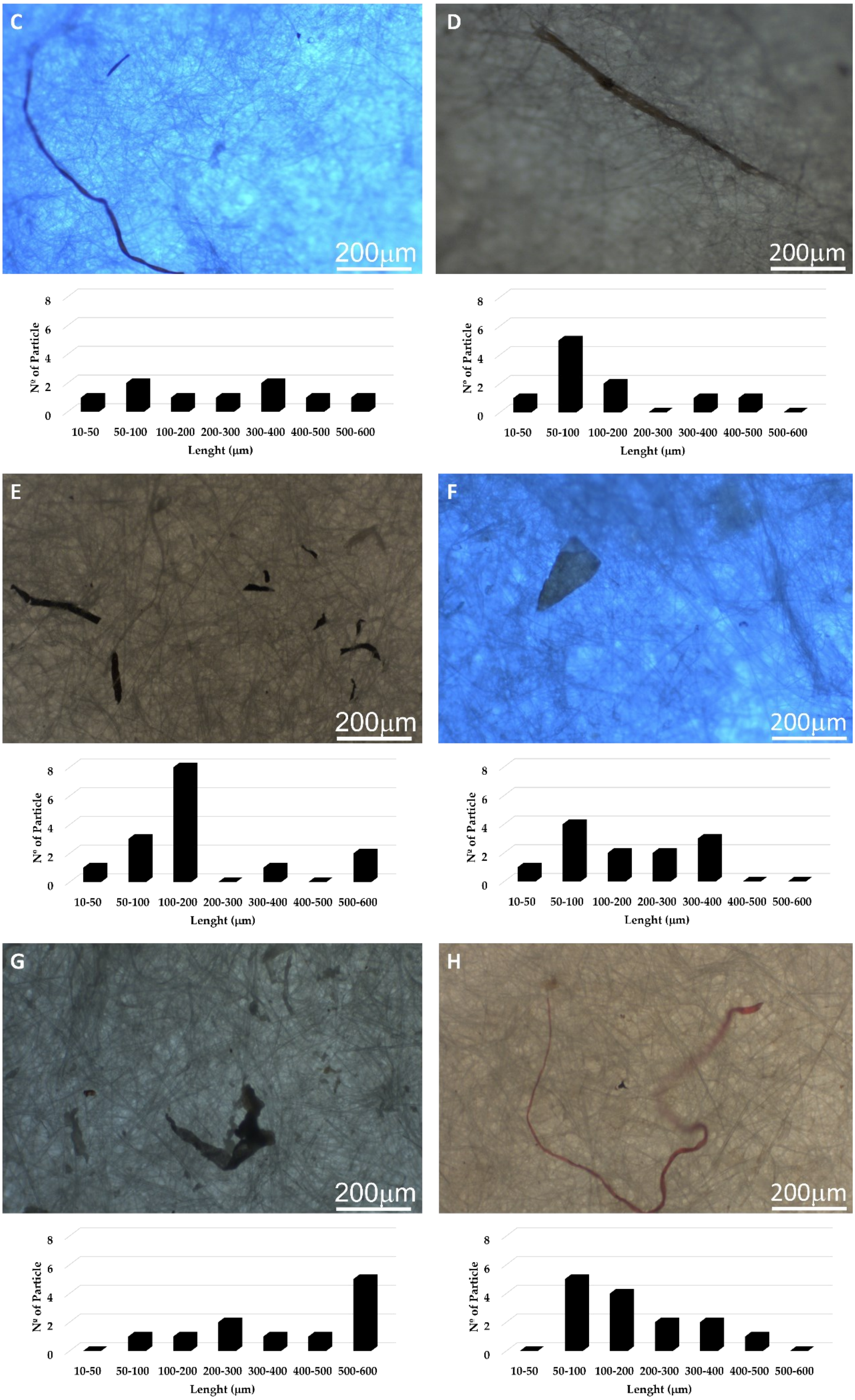
C
200μm
D
200μm
E
200μm
F
200μm
G
200μm
H
200μm
Nº of Particle
0
2
4
6
8
10-50
50-100
100-200
200-300
300-400
400-500
500-600
Lenght (μm)

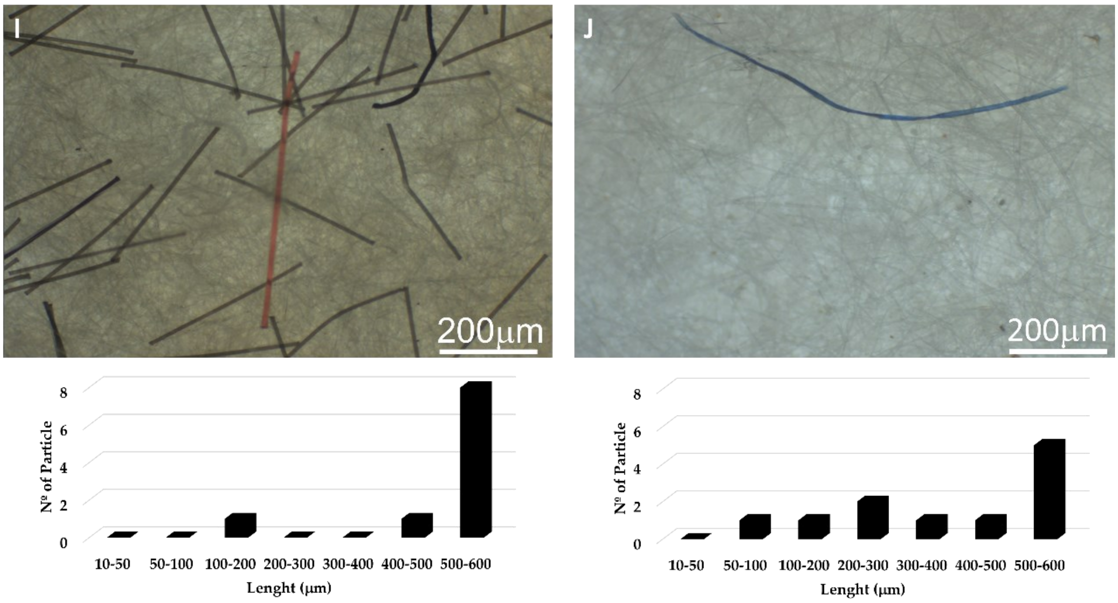

Figure S1: Optical micrographs and size distributions of the MPs before (left column) and after (right column) applying the cleaning procedure developed in this work to the resin (A,B), paint (C,D), pharmaceutical (E,F), PVC (G,H) and textile (I,J) effluents. Note that below each representative optical micrograph the correspondent size distributions can be found.

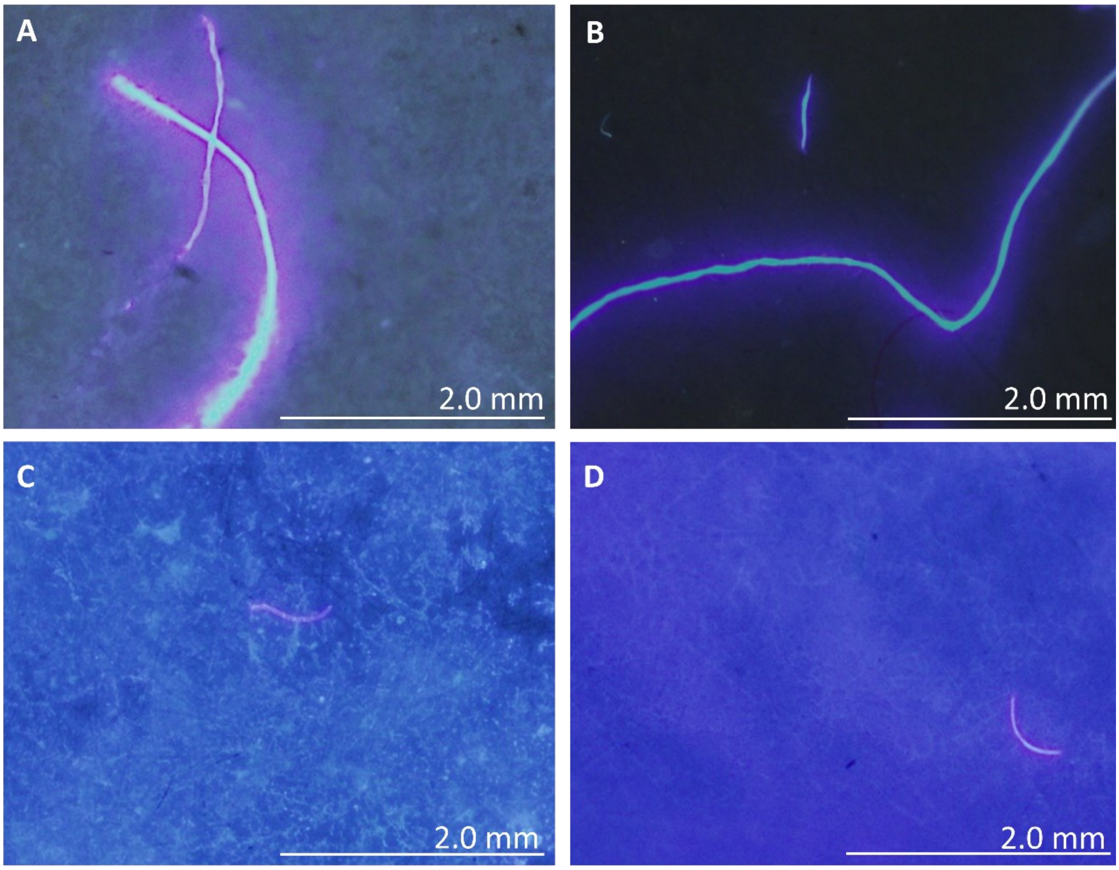

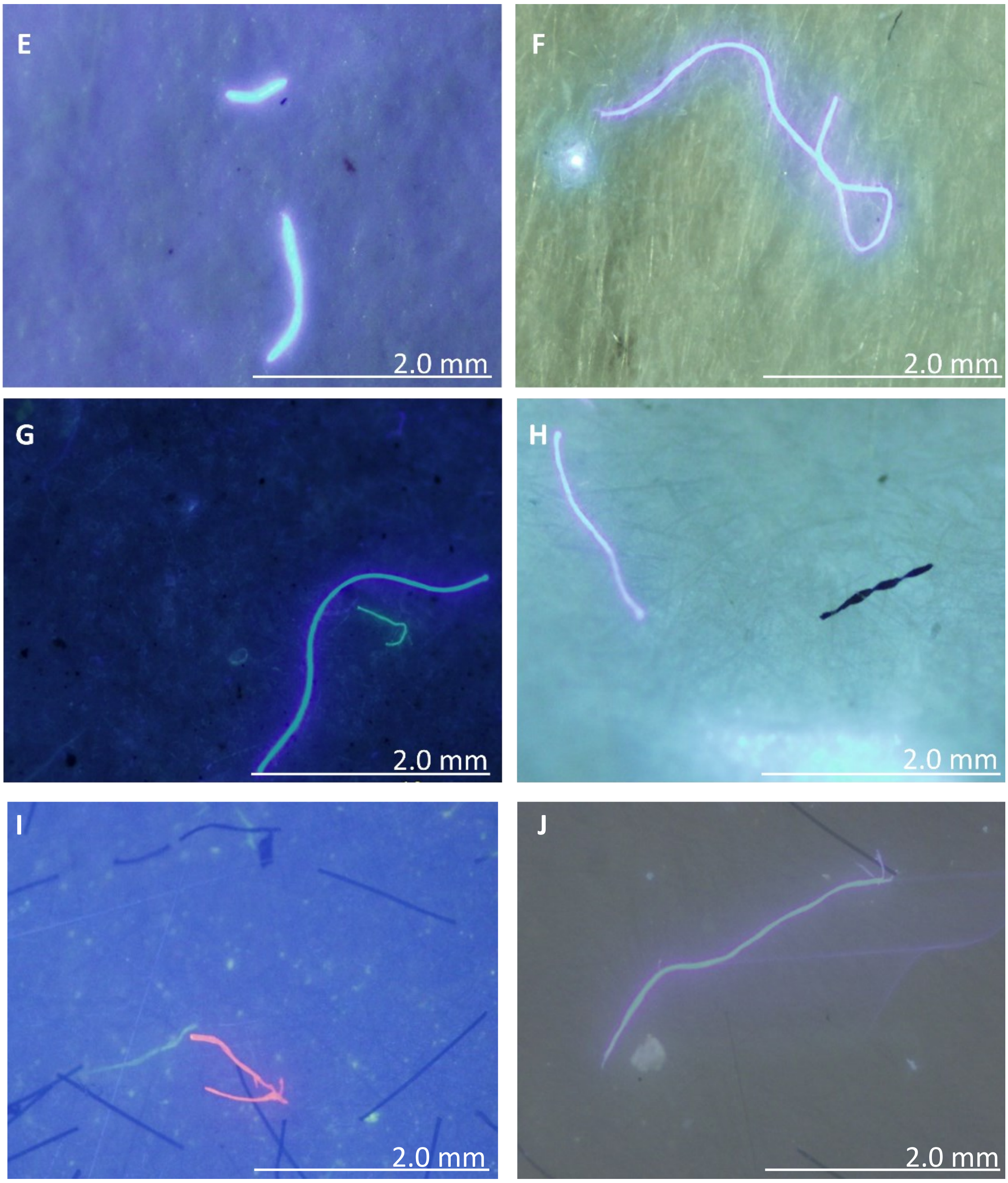

Figure S2: Fluorescent micrographs of the MPs before (left column) and after (right column) applying the cleaning procedure developed in this work to the PVC (A,B), resin (C,D), paint (E,F), pharmaceutical (G,H) and textile (I,J) effluents.

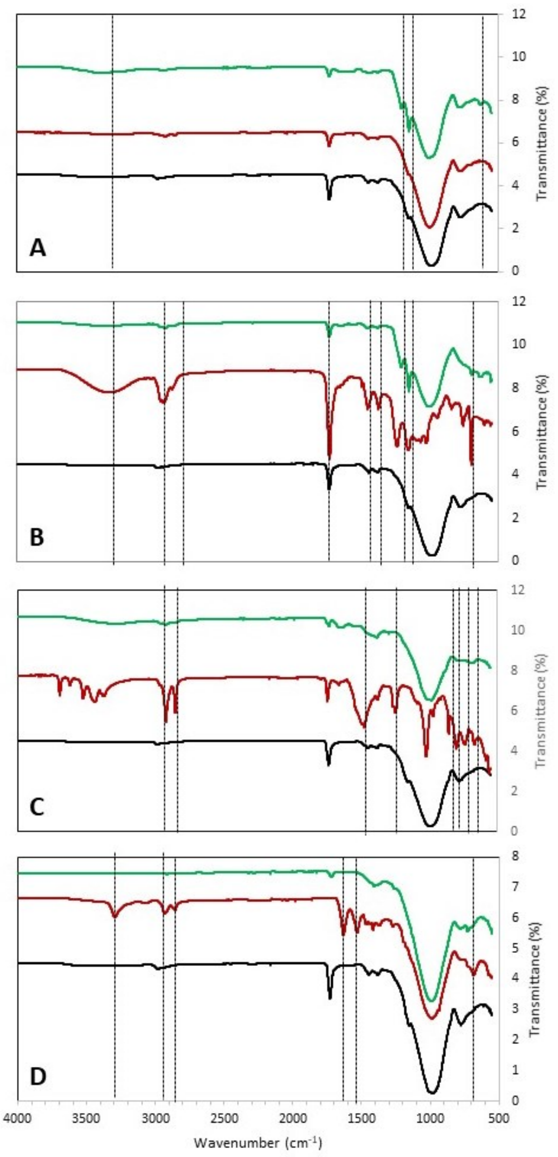

Figure S3: FTIR spectra of the MPs from the (A) resin, (B) paint, (C) PVC and (D) textile effluents before (red curves) and after (green curves) the in-house WWTP. The FTIR spectra from the filter (black curve) is also shown. The main vibrational modes are highlighted with the dashed vertical lines and its assignment is discussed in the main text and bellow.

In the resin effluent (Figure S3A), the band at 731-720 cm^{-1} is assigned to the rocking deformation of PE, while the band at 1147-1201 cm^{-1} corresponds to the wagging deformation. The band at 3400 cm^{-1} is assigned to the OH groups. In the paint effluent (Figure S3B), more vibrational bands can be observed in addition to the bands at 731-720 cm^{-1} and 1100-1200 cm^{-1} previously described. In particular, the band at 1400–1500 cm^{-1} can be assigned to the bending deformation of CH_2, while the band at 2840–3000cm^{-1} is assigned the CH_2 asymmetric stretching mode also

characteristic of PE [1]. The textile effluent (Figure S3D) presents a FTIR spectrum similar to the pharmaceutical effluent (Figure 5, main text), where the main rocking and wagging deformations and CH_2 asymmetric stretching of PE can be identified as well. The FTIR spectrum of the PVC effluent shows peaks at 2972 cm-1 and 2910cm-1, consisting of the CH2 asymmetric stretching vibration mode, the peak at the higher wavenumber shows the asymmetric stretching bond of C-H and the lower peak is for the symmetrical stretching bond of C-H. The peaks around 1400 cm-1 are assigned to the C-H aliphatic bending bond. The peak at 1250 cm-1 is attributed to the bending bond of C-H near Cl. The C-C stretching bond of the PVC backbone chain occurs in the range 1000–1100 cm-1. Finally, peaks in the range of 600-650cm-1 correspond to the C-Cl gauche bond [2] .

Reference

(1) Gulmine, J. V.; Janissek, P. R.; Heise, H. M.; Akcelrud, L. Polyethylene Characterization by FTIR. *Polymer Testing* **2002**, *21* (5), 557–563. https://doi.org/10.1016/S0142-9418(01)00124-6.

(2) Pandey, M.; Joshi, G. M.; Mukherjee, A.; Thomas, P. Electrical Properties and Thermal Degradation of Poly(Vinyl Chloride)/Polyvinylidene Fluoride/ZnO Polymer Nanocomposites: PVC/PVDF/ZnO Polymer Nano-Composites. *Polym. Int.* **2016**, *65* (9), 1098–1106. https://doi.org/10.1002/pi.5161.
